# Efficacy, safety and effectiveness of licensed rotavirus vaccines: a systematic review and meta-analysis for Latin America and the Caribbean

**DOI:** 10.1186/s12887-016-0771-y

**Published:** 2017-01-13

**Authors:** Raúl F. Velázquez, Alexandre C. Linhares, Sergio Muñoz, Pamela Seron, Pedro Lorca, Rodrigo DeAntonio, Eduardo Ortega-Barria

**Affiliations:** 1 Unidad de Investigación Médica en Enfermedades Infecciosas, Hospital de Pediatría, Centro Médico Nacional Siglo XXI, Instituto Mexicano del Seguro Social, Ciudad de México, México; 2 Instituto Evandro Chagas, Secretaria de Vigilância em Saúde, Virology Section, Av. Almirante Barroso 492, 66.090-000 Belém, Pará Brazil; 3 Centro de Excelencia Capacitación, Investigación y Gestión para la Salud basada en Evidencias CIGES, Universidad de La Frontera, Temuco, Chile; 4 GSK Vaccines, Panamá City, Panamá

**Keywords:** Rotavirus, Gastroenteritis, Diarrhea, Vaccination, RV5, RV1, Efficacy, Safety, Effectiveness, Hospitalization

## Abstract

**Background:**

*RotaTeq™* (RV5; Merck & Co. Inc., USA) and *Rotarix™* (RV1, GlaxoSmithKline, Belgium) vaccines, developed to prevent rotavirus diarrhea in children under five years old, were both introduced into national immunization programs in 2006. As many countries in Latin America and the Caribbean have included either RV5 or RV1 in their routine childhood vaccination programs, we conducted a systematic review and meta-analysis to analyze efficacy, safety and effectiveness data from the region.

**Methods:**

We conducted a systematic search in PubMed, EMBASE, Scielo, Lilacs and the Cochrane Central Register, for controlled efficacy, safety and effectiveness studies published between January 2000 until December 2011, on RV5 and RV1 across Latin America (where both vaccines are available since 2006). The primary outcome measures were: rotavirus-related gastroenteritis of any severity; rotavirus emergency department visits and hospitalization; and severe adverse events.

**Results:**

The results of the meta-analysis for efficacy show that RV1 reduced the risk of any-severity rotavirus-related gastroenteritis by 65% (relative risk (RR) 0.35, 95% confidence interval (CI) 0.25; 0.50), and of severe gastroenteritis by 82% (RR 0.18, 95%CI 0.12; 0.26) versus placebo. In trials, both vaccines significantly reduced the risk of hospitalization and emergency visits by 85% (RR 0.15, 95%CI 0.09; 0.25) for RV1 and by 90% (RR 0.099, 95%CI 0.012; 0.77) for RV5. Vaccination with RV5 or RV1 did not increase the risk of death, intussusception, or other severe adverse events which were previously associated with the first licensed rotavirus vaccine. Real-world effectiveness studies showed that both vaccines reduced rotavirus hospitalization in the region by around 45–50% for RV5 (for 1 to 3 doses, respectively), and, by around 50–80% for RV1 (for 1 to 2 doses, respectively). For RV1, effectiveness against hospitalization was highest (around 80–96%) for children vaccinated before 12 months of age, compared with 5–60% effectiveness in older children. Both vaccines were most effective in preventing more severe gastroenteritis (70% for RV5 and 80–90% for RV1) and severe gastroenteritis (50% for RV5 and 70–80% for RV1).

**Conclusion:**

This systematic literature review confirms rotavirus vaccination has been proven effective and well tolerated in protecting children in Latin America and the Caribbean.

**Electronic supplementary material:**

The online version of this article (doi:10.1186/s12887-016-0771-y) contains supplementary material, which is available to authorized users.

## Background

Diarrheal diseases are the second most common cause of mortality in children under five years of age [1]. Indeed, an estimated 2.5 billion children suffer from diarrheal diseases and 1.5 million children die worldwide from diarrhea every year. Most cases occur in developing nations [1]. The most common etiological agent of acute infectious diarrhea in children under five years old is rotavirus [2]. In fact, approximately one third of fatal diarrheal cases, estimated in 2008 as 453,000 children per year, mostly in less developed countries [3] and 40% of hospital admissions, due to diarrhea among children under five years of age, were caused by rotaviruses [1]. Severe rotavirus gastroenteritis is largely limited to children aged 6–24 months. Additionally, in developing countries, three-quarters of children suffer their first rotavirus diarrhea episode before 12 months of age [4]. Reinfections are common as mild diarrhea or asymptomatic infections [5]. Several studies have shown that immunization helps to reduce the number of diarrhea-associated deaths by preventing rotavirus infections or by reducing their severity [6].

The first licensed rotavirus vaccine was *RotaShield™* (Wyeth Laboratories, Inc., Marietta, Pennsylvania, USA), with 80–100% efficacy in preventing severe rotavirus diarrhea in randomized clinical trials [7–9]. Although licensed for routine use in the United States in 1998, it was soon withdrawn from the market due to an increased risk of intussusception, estimated at 10–20 cases per 100,000 doses [10–12]. Two new rotavirus vaccines with different antigen compositions and dosing schedules have been approved for human use since 2006 in several countries, including 17 developing countries in Latin America and the Caribbean region [13, 14], where an estimated 88 deaths per 100,000 children under 5 years occur annually [15]. RV5 (*RotaTeq™*; Merck & Co., Inc., West Point, PA, USA) is a three-dose oral pentavalent (G1, G2, G3, G4, P8) bovine-human reassortant vaccine, administered at 6–12 weeks of age, with a gap of 4–10 weeks between subsequent doses. RV1 (*Rotarix™* RIX4414; GlaxoSmithKline, Belgium), is a two-dose oral monovalent human attenuated vaccine derived from a G1[P8] virus [4], administered at 8 and 16 weeks of age. The WHO recommended both vaccines for routine child immunization globally, based on trial results [16–18], with surveillance and long term monitoring for intussusception and other potential health problems [19].

The aim of the present work was to conduct a systematic review and meta-analysis on the efficacy, safety, and effectiveness of RV5 and RV1 in Latin America and the Caribbean. These analyses will benefit from the early introduction of the vaccine in these developing nations where mortality from rotavirus disease is highest [20]. Vaccine effectiveness studies provide real world data on outcomes and safety, and, meaningful long term public health data. The findings will be useful to guide decision-making with respect to the continuation, adjustment and expansion of rotavirus vaccine programs in developing countries.

## Methods

We carried out a systematic review and meta-analysis to describe, compare and summarize the vaccine efficacy, from pre-licensure randomized clinical trials, and vaccine effectiveness, from post-licensure comparative observational studies, of RV5 and RV1, in preventing rotavirus gastroenteritis and reducing hospitalization and emergency visits across Latin American countries, where both vaccines have been available for the last decade. In addition, safety data of RV5 and RV1 were collected to assess the risk of intussusception, severe adverse events or death potentially associated with vaccination. We followed the Preferred Reporting Items for Systematic Reviews and Meta-Analyses, PRISMA Statement [21] in the conduct of this review.

### Data collection and analysis

#### Database search strategy

We conducted a sensitive and systematic search in the following electronic databases: PubMed, EMBASE, Scielo, Lilacs and the Cochrane Central Register for Controlled Trials. We used the free and Medical Subject Heading (MeSh) search terms, Boolean operators, time limits and methodological filters available on each database. The search strategy is fully described in Additional file 1: web-appendix 1 of the supplementary material. Articles published between January 2000 until December 2011 were considered in the review and no language limitation was applied.

#### Study screening and data extraction

After selecting the records, three independent reviewers applied inclusion criteria to assess the eligibility of abstracts and full-text papers, according to the settings shown in Additional file 1: web-appendix 2. Briefly, for the efficacy and safety evaluation, only randomized clinical trials including an experimental group receiving RV5 or RV1 were included. Case–control studies evaluating effectiveness were included if one group was exposed to either licensed vaccine. The evaluated population exclusively included children under five years old from Latin America and the Caribbean region. The primary outcome measures included: rotavirus-related gastroenteritis of any severity; emergency department visits and hospitalization due to rotavirus; and severe adverse events (see Additional file 1: web-appendix 2). Rotavirus gastroenteritis severity was based on the Vesikari Clinical Severity Scoring System that includes assessment of diarrhea, vomiting, temperature, dehydration and treatment [22, 23]. The scale, from 0 to 20, was used to relate to severity as such; scores above 11 were considered ‘severe’, and above 19 were considered ‘more severe’, as in a previous study.

Reviewers used a standard eligibility form based on the inclusion criteria. Publications that were duplicate or described studies that did not fulfill the inclusion criteria, as well as editorials were excluded from the analysis. Reviewers collected data on vaccine type and dose, number of participants in each group, dropouts or withdrawals, duration of follow-up, type of population and frequency of the defined outcome on pre-tested data extraction sheets (Additional file 1: web-appendix 3). When the reviewers disagreed about the evaluation of eligibility, either a fourth reviewer was consulted or a re-evaluation was done until consensus was achieved.

#### Assessment of the risk of bias in included studies

For the efficacy and safety evaluation, two independent and masked reviewers assessed the risk of bias of the included studies, according to the Cochrane Collaboration criteria [24]. These criteria consider: sequence generation, blinding of participants and personnel, blinding of outcome, data integrity, and selective outcome reporting. Reviewers used a standard form for risk of bias evaluation (see Additional file 1: web-appendix 4). A judgment about the summary risk of bias per study was made (see Additional file 1: web-appendix 6) based on the individual bias assessments within each study. Disagreements were solved by consensus.

### Statistical analysis

Efficacy of the vaccines was defined as the relative risk reduction calculated as (1− relative risk) × 100, obtained from data corresponding to randomized clinical trials. However, since the meta-analysis was performed with relative risk, the forest plots and the description of the results are presented as the calculated % efficacy and the estimated relative risk with the calculated 95% Confidence Intervals (95% CI). For studies not included in the meta-analysis, only the percentage of efficacy (95% CI) is presented. For safety, the strength of association between rotavirus immunization and a) intussusception, b) severe adverse events, and c) mortality caused or associated to vaccination was assessed by calculating the relative risk and 95% CI. Effectiveness was reported as (1–Odds Ratio) x 100 in the case–control studies.

Summary relative risk was calculated from the meta-analysis. A fixed-effect model (Mantel-Haenszel method [25]), assuming trial homogeneity, and a random-effects model (DerSimonian and Laird method [26]), accounting for trial heterogeneity, were used.

Results were reported with the random-effects model if there were differences between trials influencing the size of the treatment effect or when heterogeneity was detected. This was only applicable to the efficacy estimate. The Chi-squared (*χ* ^2^) test was applied to determine heterogeneity (*p* <0.10 was considered significant) and the I^2^ statistic to quantify inconsistency across trials (I^2^ > 50% indicated heterogeneity). On the contrary, for effectiveness estimates, only summary figures are presented since there were differences in study designs making it difficult for the individual study to meet the criteria to be eligible for the meta-analysis. Analyses were performed using Statistical Analysis System 9.0 (SAS, SAS Institute, Cary, NY, USA).

## Results

### Efficacy and safety assessment

#### Study selection

**Fig. 1:**
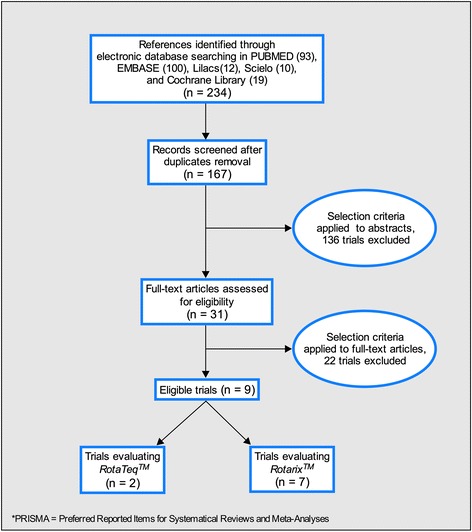
PRISMA flow chart: rotavirus vaccine efficacy and safety. Combined PRISMA* flow chart for the systematic review to evaluate rotavirus vaccine efficacy and safety in countries from Latin America and the Caribbean

Nine out of the 234 reviewed citations fulfilled the eligibility criteria described in Additional file 1: web-appendix 2. The selection of the included literature is depicted as a flow diagram in Fig. 1 (for detailed information of the included studies see Additional file 1: web-appendix 5). Data from five studies were included from the original publications as well as from four subsequent publications focusing on specific subsets of countries within the global trials, or on longer term follow up data.

#### Risk of bias of included studies

The assessment of partiality during the selection of the literature indicated that blind assignment and outcome were the main bias sources in this study. The bias risk summary of the nine evaluated studies was low in 22% and moderate in 78% of the cases (see Additional file 1: web-appendix 6).

#### Description of selected studies

Two publications evaluated RV5 from one large trial of 4,489 participants [27] and a sub-study of the trial in 1,650 children in Jamaica (high quality evidence) [28]. Seven publications assessed RV1 in 26,342 children from four original trials [17, 18, 29, 30] and 15,326 children from three sub-studies of these trials (high quality evidence) [31–33].

Studies were conducted in 15 different countries. For RV1: Brazil (5), Colombia and Mexico (4), Argentina, Dominican Republic, Honduras, Panama and Venezuela (3), Chile and Nicaragua (2) and Peru (1); for RV5: Jamaica (2) and Costa Rica, Guatemala, Mexico and Puerto Rico (1).

General descriptive information concerning the type of rotavirus vaccine, vaccination schedule and dose, location, population size, duration of follow-up, participants’ age and outcome is provided in Additional file 1: web-appendix 7.

#### Efficacy of rotavirus vaccines

##### Rotavirus vaccination reduced hospital admissions and emergency department visits

**Fig. 2:**
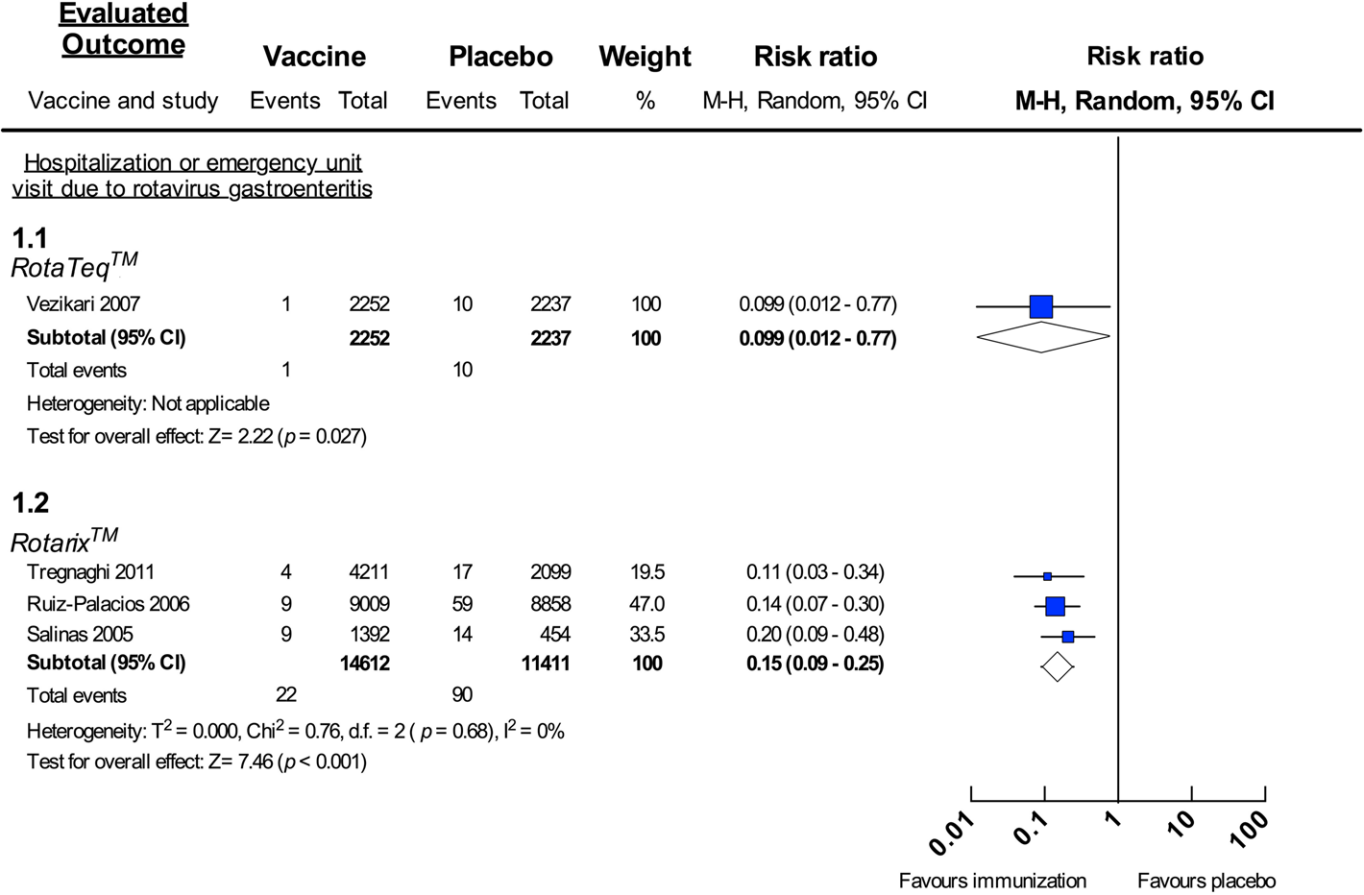
Forest plot: Hospitalization or emergency visits for rotavirus gastroenteritis in trials. Forest plot of meta-analysis for hospitalization or emergency unit visit due to rotavirus gastroenteritis in trials. Rotavirus vaccination vs. placebo: relative risk for requiring hospitalization or emergency unit visit due to rotavirus gastroenteritis

A pre-licensure study across several regions assessed the efficacy of RV5 based on the combined reduction of hospitalizations and visits to the emergency department associated with rotavirus gastroenteritis. Within the Latin America and Caribbean region, the study described 90% efficacy of RV5 (relative risk 0.099, 95% CI: 0.012–0.77, 4,489 participants, one trial, Analysis 1.1 in Fig. 2) [27]. In a sub-study of Jamaican children, not included in meta-analysis because they were evaluated globally in the larger trial, a reduction of 82.2% (95% CI: 15.1 to 98) in hospitalizations, or emergency department visits attributable to rotavirus gastroenteritis involving any serotype, was found after three doses of RV5 [28].

Pooled data from three pre-licensure studies [17, 18, 30] showed that during one year of follow up, RV1 reduced hospital admissions due to severe rotavirus gastroenteritis by 85% (relative risk 0.15, 95% CI: 0.09–0.25, 26,023 participants, Analysis 1.2 in Fig. 2). A similar efficacy percentage was reported for RV1 in preventing rotavirus-related gastroenteritis hospitalizations in two additional sub-studies (not included in this meta-analysis) conducted across Latin America (83%; 95% CI: 73.1–89.7) [33] after a 2-years follow up, and in Brazil (80.3%; 95% CI: 51.1–92.5) [31].

##### Rotavirus vaccination decreased both diarrhea of any cause and rotavirus-related gastroenteritis

**Fig. 3:**
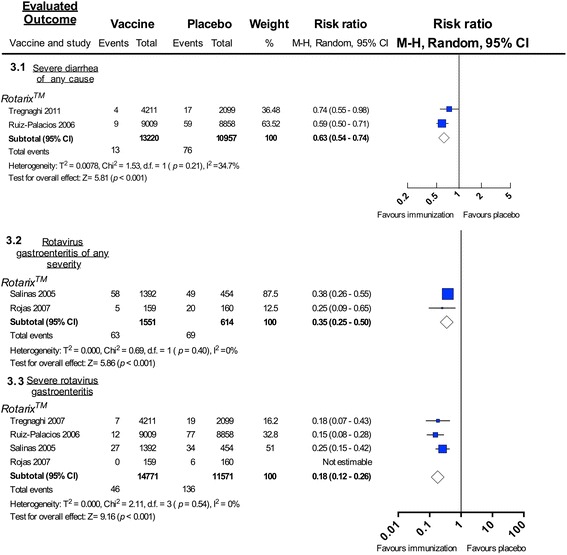
Forest plot: Severe diarrhea, rotavirus gastroenteritis (any severity) and severe rotavirus gastroenteritis in trials. Forest plot of meta-analysis for preventing rotavirus gastroenteritis of any severity in trials. Rotavirus vaccination vs. placebo: relative risk for protecting against rotavirus gastroenteritis of any severity or severe rotavirus gastroenteritis

RV1 reduced the occurrence of diarrhea of any cause by 37% in vaccinated children as compared to those receiving placebo, during the first year following vaccination (relative risk 0.63; 95% CI: 0.54–0.74; 24,177 participants, 2 trials, Analysis 3.1 in Fig. 3) [18, 30]. A sub-study, not included in this meta-analysis, reported a similar result but during the second year of follow up (efficacy 39%; 95% CI: 30.1–46.9) [33]. RV1 reduced the overall presentation of not only diarrheal disease, but specifically for rotavirus gastroenteritis of any severity by 65% (relative risk 0.35; 95% CI: 0.25–0.50; 2,165 participants, 2 trials, Analysis 3.2 in Fig. 3) [18, 30]. This percentage differed from sub-studies not included in the meta-analysis and conducted in Mexico (76.3%; 95% CI: 48.9–89.3) [32] and Brazil (43.5%; 95% CI: 48.9–89.3) [31]. Finally, RV1 reduced the frequency of severe rotavirus gastroenteritis by 82% (relative risk 0.18; 95% CI: 0.12–0.26; 26,342 participants, 4 trials, Analysis 3.3 in Fig. 3) [17, 18, 29, 30]. This estimate was close to that reported for Latin American children after a two year-follow up (79%; 95% CI: 66.4–87.4) [33] and Mexican children (90%; 95% CI: 66.4–87.4) [32], but greater than the one reported in Brazil (64.5%; 95% CI: 30.7–81.7) [29]; once again these three sub-studies were not included in the meta-analysis since they were evaluated in a previous larger trial.

#### Safety of rotavirus vaccines

##### Rotavirus vaccination did not increase the risk of death, intussusception or other severe adverse events

Safety evaluations were not frequently reported for the pre-licensure studies included in this meta-analysis (see Additional file 1: web-appendix 7). Where reported, RV1 did not increase the risk of death across the vaccinated children (relative risk 1.34; 95% CI: 0.92–1.96; 71,690 participants, 3 trials, Analysis 4.1 in Additional file 1: web-appendix 9) [17, 18, 30]. Similarly, in the Jamaican trial with RV5, none of the four deaths (1 vaccinated infant and 3 placebo recipients) were vaccine-related [28].

Pooled data for RV1 showed no increased risk of intussusception among vaccinated children (relative risk 0.64; 95% CI: 0.31–1.34; 71,690 participants, 3 trials, Analysis 5.1 in Additional file 1: web-appendix 10) [17, 18, 30]. In the RV5 studies, there was only one confirmed case of intussusception in a RV5 recipient, compared to three cases in the placebo group [28].

Three pre-licensure studies evaluated the association of RV1 with severe adverse events [17, 18, 30]; a list of the most frequent severe adverse events reported in these studies is presented in Additional file 1: web-appendix 11. The pooled results indicated no increased risk in the occurrence of severe outcomes in RV1-immunized children as compared to controls (relative risk 0.89; 95% CI: 0.83–0.95; 71,690 participants, 3 trials, Analysis 6.1 in Additional file 1: web-appendix 12) [17, 18, 30]. Only one study evaluated severe adverse events and the use of RV5 [28]. In this study, severe adverse outcomes were reported in 3.5% (31/898) and 4.8% (43/904) vaccinated or placebo exposed children, respectively. Only single cases of febrile infection and gastroenteritis were associated with RV5.

### Effectiveness and impact assessment

#### Study selection

**Fig. 4:**
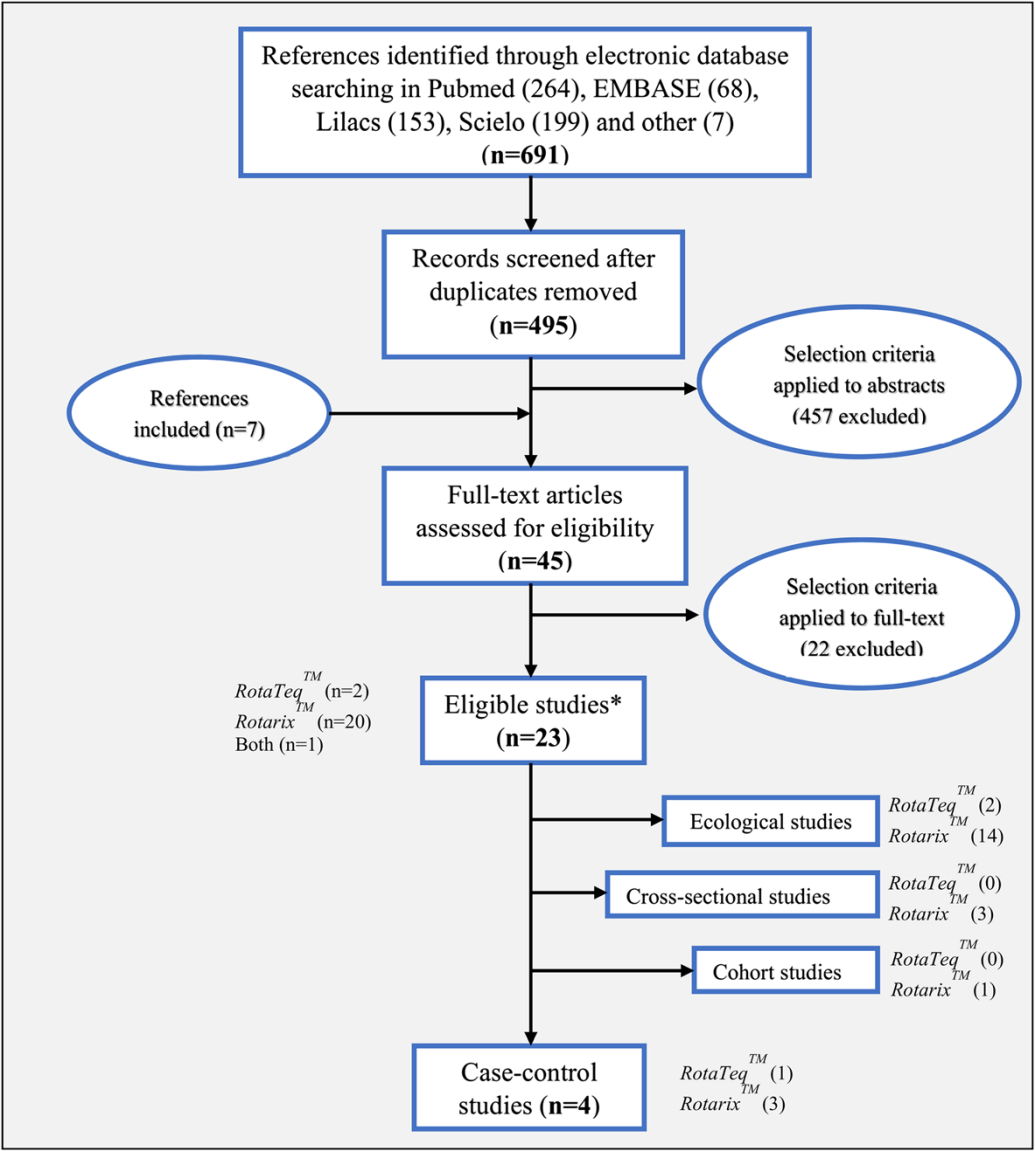
PRISMA flow chart: rotavirus vaccine effectiveness. PRISMA* flow chart for the systematic review to evaluate rotavirus vaccine effectiveness in countries from Latin America and the Caribbean

Of the 691 citations identified, 45 full text articles were screened and 23 were identified for analysis. Of the 23 publications, four case–control studies assessing vaccine effectiveness were included for analysis. The remaining studies used various methods to assess the impact of vaccination (see Fig. 4, Additional file 1: web-appendix 2 and 13 for details).

#### Description of selected studies

The effectiveness of RV5 was evaluated in one study in Nicaragua, and effectiveness of RV1, in two studies in Brazil and one in El Salvador. Both, the summary of the characteristics and the main results of these studies stratified by design and vaccine type are presented in Additional file 1: web-appendix 14 and 15, respectively.

#### Effectiveness and impact of rotavirus vaccines

##### Rotavirus vaccines reduced the likelihood of rotavirus infection, gastroenteritis-related hospitalization and death in children under five years of age

**Fig. 5:**
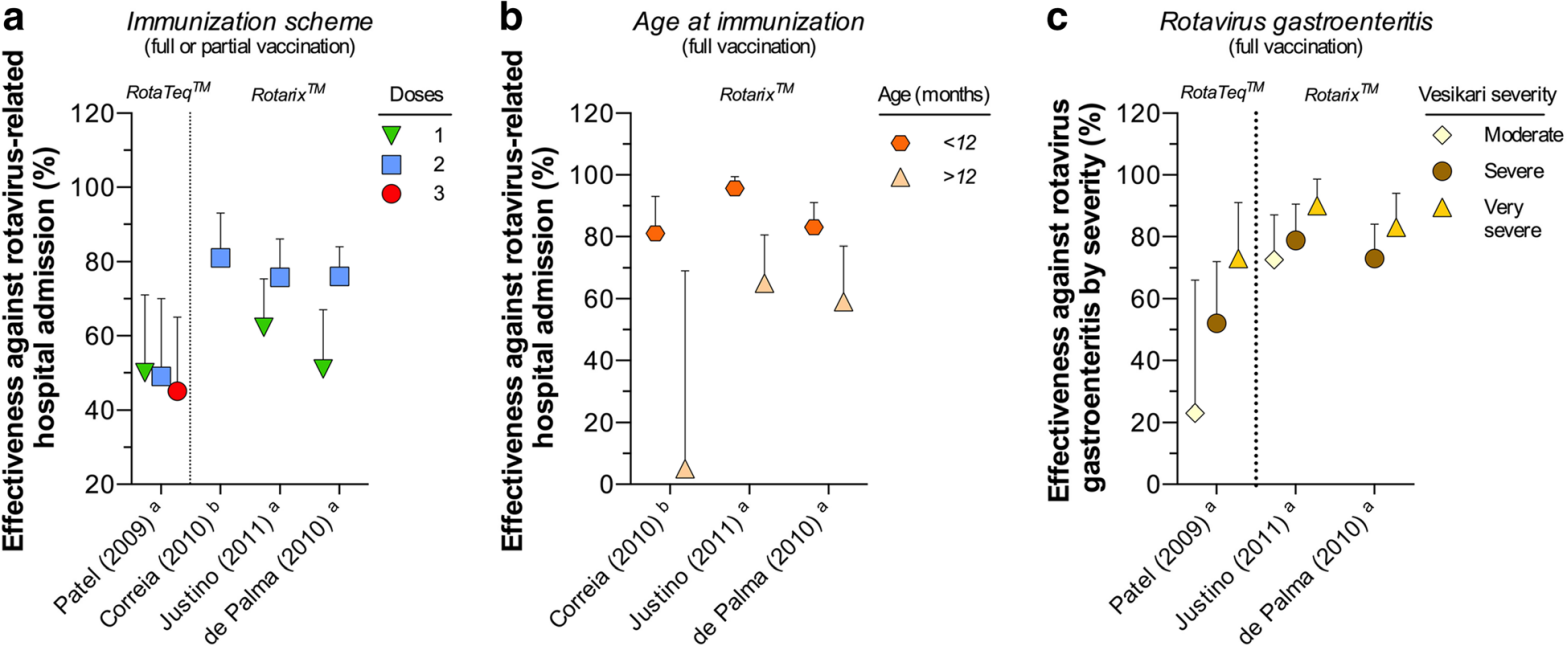
Effectiveness of *RotaTeq* ^™^ and *Rotarix* ^™^ vaccines in preventing rotavirus-related hospital admissions or rotavirus gastroenteritis. Effectiveness of *RotaTeq* ^™^ and *Rotarix* ^™^ vaccines in preventing rotavirus-related hospital admissions or rotavirus gastroenteritis according to (**a**) partial or full vaccination scheme, (**b**) age at immunization, and (**c**) severity of the disease. ^a^ Neighborhood controls, ^b^ Rotavirus negative control participants

Four case–control studies assessed the effectiveness of RV1 (3) and RV5 (1) against severe rotavirus gastroenteritis and hospitalizations/emergency visits due to rotavirus [34–37]. Effectiveness was evaluated by comparing: immunization scheme (partial vs. full-dose administration), age at immunization (<12 months vs. >12 months of age) or the reduction in the severity of the rotavirus-related gastroenteritis (Fig. 5).

One study evaluating RV5 in children from Nicaragua [34] showed similar vaccine effectiveness against hospitalization when partial or full three-dose schedules were administered (range from 45 to 50%, Fig. 5a). However, for subjects fully vaccinated with RV5, effectiveness against moderate, severe and more severe rotavirus-related episodes of diarrhea increased according to disease severity (23, 52 and 73% effectiveness, respectively; Fig. 5c).

RV1 effectiveness against hospitalization was highest when administered under a two-dose scheme (range from 75.8 to 81%, Fig. 5a), as well as when full vaccination was administered to children under the age of twelve months (range from 81 to 95.7%, Fig. 5b) [35–37]. If administered according to a one-dose scheme, the effectiveness of RV1 in preventing hospital admission due to rotavirus ranged from 51 to 62.3% (Fig. 5a). However, the protecting effect diminished in vaccinated children over one year of age (range from 5 to 65.1%, Fig. 5b). Additionally, full immunization with RV1 increased the effectiveness against presenting more severe rotavirus gastroenteritis (range from 83 to 90%) in comparison to less severe rotavirus-related gastroenteritis (Fig. 5c).

This review also identified a number of studies with different designs (i.e., cohort, cross-sectional and ecological) which assessed the impact of vaccination in the population in different ways. These will be the subject of a separate publication.

## Discussion

RV5 and RV1 were first introduced to the immunization programs of several developed and developing countries in the last decade. This seemed to be the ideal time to conduct a systematic review and meta-analysis to evaluate the long-term benefits and impact of implementing massive vaccination programs with both vaccines in Latin America, from the period when the vaccines were first introduced. We gathered published information on the efficacy, safety and effectiveness of both vaccines in Latin American and Caribbean children between 2000 and 2011. Having conducted a thorough selection process and literature analysis, we conclude that RV5 and RV1 have significantly reduced hospital admissions and emergency department visits, the frequency of diarrheal disease of any cause and rotavirus-related gastroenteritis and the likelihood of children of getting infected by rotavirus over time. Pre-licensure studies with RV5 or RV1 did not show an increase in the frequency of intussusception and other severe adverse events, previously associated with rotavirus immunization. Vaccination did not increase the risk of death among children. In general, protection against rotavirus gastroenteritis was greater if vaccination occurred during the first year of life and was administered according to the recommended schedule and doses. Hence, over the last decade, vaccination with RV5 and RV1 has proven to be effective*,* safe, and efficient in protecting children under five years of age across Latin America and the Caribbean.

Several recent studies suggest that RV1 and RV5 could be associated with a slight increase in the risk of developing intussusception. Both RV1 and RV5 were associated with approximately 1 to 6 excess cases of intussusception per 100,000 recipients following the first dose in Mexico, the United States and Australia [38–40]. A smaller proportion was detected after the second vaccine dose in Brazil [41]. Nevertheless, this estimate is still several times lower than the risk of intussusception reported for *RotaShield™* [42, 43]. On the other hand, a study from Germany reports an increased risk of intussusception in infants only if the first dose of rotavirus vaccine is administered after 90 days of age [44]. For this reason, some authors [45] have proposed a re-evaluation of the age-limit for the administration of the first dose of vaccine from 16 weeks to the original 12 week age-limit recommended by manufacturers. Conversely it can be argued that there is insufficient evidence to suggest that the risk of intussusception is lower in children vaccinated at an earlier age and that an extended vaccination window may increase vaccine coverage and its benefits, especially in developing countries where not all of the children receive vaccination according to the recommended dosing schedules [45]. Taking all these considerations into account it was estimated that the benefits of rotavirus vaccination against diarrhea hospitalizations and death from rotavirus infection far exceeded the risk of intussusception [46]. Hence, WHO has recommended keeping the rotavirus vaccines in all national immunization programs worldwide [19].

At the present time, 19 countries and territories in Latin America and the Caribbean include rotavirus vaccines in their national immunization programs [47]. Most use RV1 [13], which therefore provides the majority of the post-marketing evidence. Many studies were conducted in Brazil and Mexico, followed by Panama, Venezuela, Nicaragua and Honduras. The vaccines’ efficacy values from clinical trials against rotavirus gastroenteritis hospitalizations were between 85 and 90% (Fig. 2); RV1 was around 80% effective against severe rotavirus gastroenteritis (Fig. 3). The overall reported effectiveness in the region against more severe rotavirus gastroenteritis was of 73% for RV5 and 83% for RV1 (Fig. 5c). The effectiveness of RV5 against severe rotavirus gastroenteritis was 52% (Fig. 5c) and the effectiveness of RV1 against rotavirus gastroenteritis hospitalizations is between 76 and 96% (Fig. 5a and b). The greatest effect was seen in children under 12 months of age, as previously observed [48–50], presumably because this represents the age group targeted for vaccination (Fig. 5b). Also, studies not included in the meta-analysis indicated a greater magnitude of effectiveness than would be expected from the proportion of vaccinated children, suggesting an indirect herd effect [51–55].

The effectiveness estimates demonstrated in this analysis and those reported in subsequent studies for Latin American and the Caribbean countries [56–58] are high and similar to the efficacy values previously observed in clinical trials. However, they are somewhat lower than those reported for developed countries, including the United States [59] and Finland [60]. This is consistent with previous reports that rotavirus vaccines are more effective against severe rotavirus gastroenteritis in sub-regions with very low or low child and adult mortality [61]. Clinical trials of oral rotavirus vaccines performed in infants have demonstrated a correlation between vaccine efficacy and the socioeconomic level. In high income settings, efficacy exceeds 90%, while in middle (as are the majority of Latin America and Caribbean countries) and low income settings the values drop to 80% [17, 18, 31] and 45% [62–65], respectively. Although the reasons for this phenomenon are unclear, a range of hypotheses has been proposed, which include immunological and epidemiologic factors including nutritional status [66, 67], concomitant infection, greater diversity of rotavirus strains circulating in many developing countries [68], as well as socioeconomic conditions affecting health care access. It has also been shown that vitamin A deficiency impairs immune responses to rotavirus vaccines in animal models [69, 70]. However, since most of the rotavirus-associated fatalities occur in low income countries [20], despite the lower vaccine efficacy, the number of severe disease cases and deaths prevented by vaccines are likely to be higher than in high income countries.

Although rotavirus vaccines were developed from the most common circulating rotavirus strains, it has been observed that they also confer protection against other strains [17, 71, 72], suggesting an important role for heterotypic protective immunity. According to this observation, both commercially available vaccines have been shown to be highly effective against severe rotavirus disease, despite one being monovalent and the other pentavalent [59, 73]. This is important because data from countries in Asia and Africa show greater strain diversity with several rotavirus types circulating simultaneously [74].

There are a few limitations of this review that should be taken into account when analyzing the findings consolidated and presented here, especially if comparisons between vaccines or between the outcomes observed in each country are to be made. Firstly, because many of the studies did not fulfill the eligibility criteria for inclusion in the meta-analysis, the final dataset comprised very few studies which were not representative of the Latin American and Caribbean region. Additionally, the type of methodology employed to determine vaccination program outcomes considered in this analysis, used the screening method to assess the vaccine effectiveness. For example, where there are discrepancies in the data reported for the same country [50]. This analysis focused on studies published between 2000 and 2011, an update to this review is warranted for further research.

Lastly, both vaccines are not equally represented in the included studies. We have data for RV5 from just 2 of the 9 efficacy studies and 1 of the 4 studies for effectiveness. This is due to the distribution of the vaccines in Latin American and Caribbean countries, where the majority of the clinical trials were conducted using RV1 vaccine and where most countries are using this vaccine in their immunization program. This prevents a fair comparison of the outcomes of each vaccine in this region. However, in countries where both vaccines are routinely used, similar efficacy and effectiveness has been reported [59, 73, 75], which is consistent with the results of this meta-analysis. Therefore, despite all the aforementioned considerations, the results obtained from this meta-analysis are consistent with other studies and provide a general panorama of the outcomes of the implementation of rotavirus vaccination in Latin America and the Caribbean region. This information is fundamental in deciding whether the vaccination programs should be continued and gives a solid foundation for considering the expansion of these programs to other developing nations.

One of the most important aspects when analyzing the viability of a vaccine program implementation is cost-effectiveness. Although cost-effectiveness ratios vary from one country to another [76], universal vaccination of infants has been demonstrated to be cost-effective for both rotavirus vaccines, especially for middle and low income settings [19, 77]. Other vaccine characteristics, such as the number of doses or the presentation, may be taken into account when selecting the most appropriate vaccine to meet the special conditions for each country.

## Conclusion

Evidence accumulated since the implementation of rotavirus vaccination in Latin America and the Caribbean allows us to conclude that the current vaccines are effective in reducing the risk of hospitalization and death due to rotavirus infection and all-cause gastroenteritis. Irrespective of the implementation plan for rotavirus vaccination, a coordinated strategy for the prevention and treatment of childhood diarrhea will also require improvements in hygiene and sanitation levels, as well as awareness of and access to oral rehydration therapy, zinc supplementation and other effective treatments. Lastly, the benefits from rotavirus vaccination greatly exceed the risk of intussusception, especially in developing regions such as Latin America. Nonetheless, it is recommended to continue monitoring in countries where rotavirus vaccines are used.

## Additional file

Additional file 1: Web-appendix 1. Search methods: detailed search strategy. **Web-appendix 2.** Criteria for trial eligibility. **Web-appendix 3.** Extraction sheet for trials assessing efficacy/safety and effectiveness/impact of rotavirus vaccine. **Web-appendix 4.** Extraction sheet for risk of bias assessment in trials evaluating efficacy/safety of rotavirus vaccine. **Web-appendix 5.** Selected studies for efficacy/safety evaluation of rotavirus vaccine. **Web-appendix 6.** Risk of bias graph. **Web-appendix 7.** Summary of the characteristics of studies included for assessing efficacy/safety of rotavirus vaccines. **Web-appendix 8.** Forest plot of meta-analysis for severe diarrhea of any cause. **Web-appendix 9.** Forest plot of meta-analysis for treatment-related mortality. **Web-appendix 10.** Forest plot of meta-analysis for treatment-related intussusception. **Web-appendix 11.** Common severe adverse events occurring in rotavirus immunized and placebo groups. **Web-appendix 12.** Forest plot of meta-analysis for treatment-related severe adverse events. **Web-appendix 13.** Identified studies for effectiveness evaluation of rotavirus vaccine. **Web-appendix 14.** Summary of the characteristics of studies included for evaluate effectivenes of rotavirus vaccine. **Web-appendix 15.** Summary of results of studies assessing effectiveness of rotavirus vaccine. (DOCX# 123 bytes)

## Abbreviations

CI: Confidence interval
WHO: World health organization
