# Supplementary material for: Efficacy, safety and effectiveness of licensed rotavirus vaccines: a systematic review and meta-analysis for Latin America and the Caribbean
Source: BMC Pediatr. 2017 Jan 13;17:14. doi: 10.1186/s12887-016-0771-y (PMC5237165; doi:10.1186/s12887-016-0771-y)
Supplement: Additional file 1: Web-appendix 1. — Search methods: detailed search strategy. Web-appendix 2. Criteria for trial eligibility. Web-appendix 3. Extraction sheet for trials assessing efficacy/safety and effectiveness/impact of rotavirus vaccine. Web-appendix 4. Extraction sheet for risk of bias assessment in trials evaluating efficacy/safety of rotavirus vaccine. Web-appendix 5. Selected studies for efficacy/safety evaluation of rotavirus vaccine. Web-appendix 6. Risk of bias graph. Web-appendix 7. Summary of the characteristics of studies included for assessing efficacy/safety of rotavirus vaccines. Web-appendix 8. Forest plot of meta-analysis for severe diarrhea of any cause. Web-appendix 9. Forest plot of meta-analysis for treatment-related mortality. Web-appendix 10. Forest plot of meta-analysis for treatment-related intussusception. Web-appendix 11. Common severe adverse events occurring in rotavirus immunized and placebo groups. Web-appendix 12. Forest plot of meta-analysis for treatment-related severe adverse events. Web-appendix 13. Identified studies for effectiveness evaluation of rotavirus vaccine. Web-appendix 14. Summary of the characteristics of studies included for evaluate effectivenes of rotavirus vaccine. Web-appendix 15. Summary of results of studies assessing effectiveness of rotavirus vaccine. (DOCX# 123 bytes) [file 12887_2016_771_MOESM1_ESM.docx]

APPENDICES

Web-appendix 1. Search methods: detailed search strategy 1

Web-appendix 2. Criteria for trial eligibility 5

Web-appendix 3. Extraction sheet for trials assessing efficacy/safety and effectiveness/impact of rotavirus vaccine 7

Web-appendix 4. Extraction sheet for risk of bias assessment in trials evaluating efficacy/safety of rotavirus vaccine 9

Web-appendix 5. Selected studies for efficacy/safety evaluation of rotavirus vaccine 10

Web-appendix 6. Risk of bias graph 13

Web-appendix 7. Summary of the characteristics of studies included for assessing efficacy/safety of rotavirus vaccines 14

Web-appendix 8. Forest plot of meta-analysis for severe diarrhea of any cause 15

Web-appendix 9. Forest plot of meta-analysis for treatment-related mortality 15

Web-appendix 10. Forest plot of meta-analysis for treatment-related intussusception 16

Web-appendix 12. Forest plot of meta-analysis for treatment-related severe adverse events 18

Web-appendix 13. Identified studies for effectiveness evaluation of rotavirus vaccine 19

Web-appendix 14. Summary of the characteristics of studies included for evaluate effectivenes of rotavirus vaccine 21

Web-appendix 15. Summary of results of studies assessing effectiveness of rotavirus vaccine 22

# Web-appendix 1. Search methods: detailed search strategy

1. Efficacy and Safety
2. Search terms
3. “Clinical Trials as Topic”[Mesh]
4. Clinical Trial
5. ‘clinical trial’/de
6. ‘controlled clinical trial’/de
7. ‘double blind procedure’/de‘randomized controlled trial’/de
8. Search terms for rotavirus-induced gastroenteritis
9. “Rotavirus infections”[Mesh]
10. Rotavirus
11. Infection
12. Gastroenteritis
13. Search terms for human rotavirus vaccines: Rotarix^TM^ and RotaTeq®
14. “RIX4414 vaccine” [Supplementary Concept]
15. “RotaTeq®” [Supplementary Concept]
16. “Rotavirus Vaccines”[Mesh]
17. Rotarix^TM^
18. RIX4414
19. RotaTeq®
20. rota teq
21. “rotavirus vaccin*’
22. ‘Rotarix ^TM^ vaccine’
23. rv5
24. rv1
25. Search limits
26. Age from 0-18 years
27. Humans
28. Date from 01/01/2000 to 31/12/2011
29. Effectiveness
30. Search terms
31. “Comparative Effectiveness Research”[Mesh]
32. “Evaluation Studies as Topic”[Mesh]
33. “Treatment Outcome”[Mesh]
34. “Program Evaluation”[Mesh]
35. “Population Surveillance”[Mesh]
36. “Epidemiological Studies”[Mesh]
37. “Case-control Studies”[Mesh]
38. “Cohort Studies”[Mesh]
39. “Outcome Assessment (Health Care)”[Mesh]
40. Impact
41. Effectiveness
42. Surveillance
43. ‘case control study’/de
44. ‘cohort analysis’/de
45. ‘comparative study’/de
46. ‘drug surveillance program’/de
47. ‘postmarketing surveillance’/de
48. ‘impact’
49. ‘effectiveness’
50. ‘surveillance’
51. ‘comparative effectiveness research’
52. ‘evaluation studies as topic’
53. ‘treatment outcome’
54. ‘program evaluation’
55. ‘population surveillance’
56. ‘epidemiologic studies’
57. ‘outcome assessment’
58. Search terms for rotavirus-induced gastroenteritis
59. “Rotavirus”[Mesh]
60. “Rotavirus Infections”[Mesh]
61. Rotavirus
62. “rotavirus infections’
63. Gastroenteritis
64. ‘rotavirus’/exp
65. ‘rotavirus infections’/exp
66. ‘rotavirus’/exp
67. ‘infection’/exp
68. ‘gastroenteritis’/exp
69. Search terms for human rotavirus vaccines: Rotarix^TM^ and RotaTeq®
70. “Rotavirus Vaccines”[Mesh]
71. “RIX4414 vaccine” [Supplementary Concept]
72. “RotaTeq®” [Supplementary Concept]
73. (“Vaccination”[Mesh]
74. “Mass Vaccination”[Mesh]
75. “Immunization”[Mesh]
76. “Immunization Programs”[Mesh]
77. Rotarix^TM^
78. RotaTeq®
79. RIX4414
80. Vaccination
81. vaccine*
82. immunization
83. “immunization programs”
84. ‘rotavirus vaccines’/exp
85. ‘rotateq’/exp
86. ‘rix4414 vaccine’
87. ‘rix4414’
88. ‘rotarix’/exp
89. ‘vaccination’/exp
90. ‘mass vaccination’/exp
91. ‘immunization’/exp
92. ‘immunization program’
93. Exclusion terms
94. “Randomized Controlled Trial”[Publication Type]
95. “Randomized Controlled Trials as Topic” [Mesh]
96. “Editorial” [Publication Type]
97. “Comment”[ Publication Type]
98. “Review”[ Publication Type]
99. “Review Literature as Topic”[Mesh]
100. “Cost-Benefit Analysis”[Mesh]
101. “Africa”[Mesh]
102. “Asia”[Mesh]
103. “Europe”[Mesh]
104. “North America”[Mesh]
105. “Oceania”[Mesh]
106. ‘controled study’/de
107. ‘double blind procedure’/de
108. ‘interview’/de
109. ‘nonhunam’/de
110. ‘phase 2 clinical trial’/de
111. ‘phase 3 clinical trial’/de
112. ‘questionnaire’/de
113. ‘randomized controlled trial’/de
114. ‘randomized controlled trials as topic’
115. ‘editorial’/de
116. ‘comment’
117. ‘review’/de
118. ‘review literature as topic’
119. ‘cost-benefit analysis’/de
120. ‘africa’
121. ‘asia’
122. europe’
123. ‘north america’
124. ‘oceania’
125. Search limits
126. Age from 0-18 years
127. Humans
128. Date from 01/01/2000 to 31/12/2011

In LILACS we used the following search strategy: “( ( “ROTAVIRUS INFECTIONS”) or “ROTAVIRUS”) or “ROTAVIRUS VACCINES” or rotarix or rotateq or rix4414 or rv5 [Words] and “HUMANS” [Limits] and not “CONTROLLOLED CLINICAL TRIAL” [Publication type]”.

In Scielo the following search strategy was used: “rotavirus OR rotarix OR rotateq OR RIX4414 or RV5 OR “Rotavirus Vaccines””

# Web-appendix 2. Criteria for trial eligibility

Inclusion criteria for trial eligibility of efficacy/safety or effectiveness assessment of rotavirus vaccine

| **EFFICACY AND SAFETY** | | | | | | | | |
| --- | --- | --- | --- | --- | --- | --- | --- | --- |
| *Trial design* | *Target population, location objective of the trial and period of evaluation* | *Treatment* | | | | *Evaluated outcomes^a^* | |  |
| Exclusively randomized clinical trials | Children aged <12 months  Trials conducted in countries from Latin America and the Caribbean  To assess efficacy and safety of Rotavirus-immunized infants  Trial conducted after year 2000 | | - Experimental group received Rotavirus vaccine (*RotaTeq^®^* or *Rotarix^TM^*) - Placebo group - Other Rotavirus vaccine - None of the others | | - Rotavirus gastroenteritis of any severity - Severe Rotavirus gastroenteritis - Rotavirus gastroenteritis requiring hospitalization - All-cause diarrhea - Emergency department visit due to diarrhea - Mortality due to rotavirus - Severe adverse event | | | |
| **EFFECTIVENESS** | | | | | | | | |
| **Study design** | **Target population, location, objective of the study and period of evaluation** | | | **Treatment and comparison of groups** | | |  | |
| Case-control studies | Children < 5 years old  Studies carried on in countries from Latin America and the Caribbean  Evaluation of effectiveness  Studies done after year 2000 | | - One group exposed to any of currently licensed Rotavirus vaccines (*RotaTeq^®^* or *Rotarix^TM^*) - One unvaccinated group - Comparison among immunized and unvaccinated groups | | - Rotavirus gastroenteritis of any severity - Severe Rotavirus gastroenteritis - Rotavirus gastroenteritis requiring hospitalization - All-cause diarrhea - Emergency department visit due to diarrhea | | | |

^a^ Included trials evaluated at least one of these outcomes

# Web-appendix 3. Extraction sheet for trials assessing efficacy/safety and effectiveness/impact of rotavirus vaccine


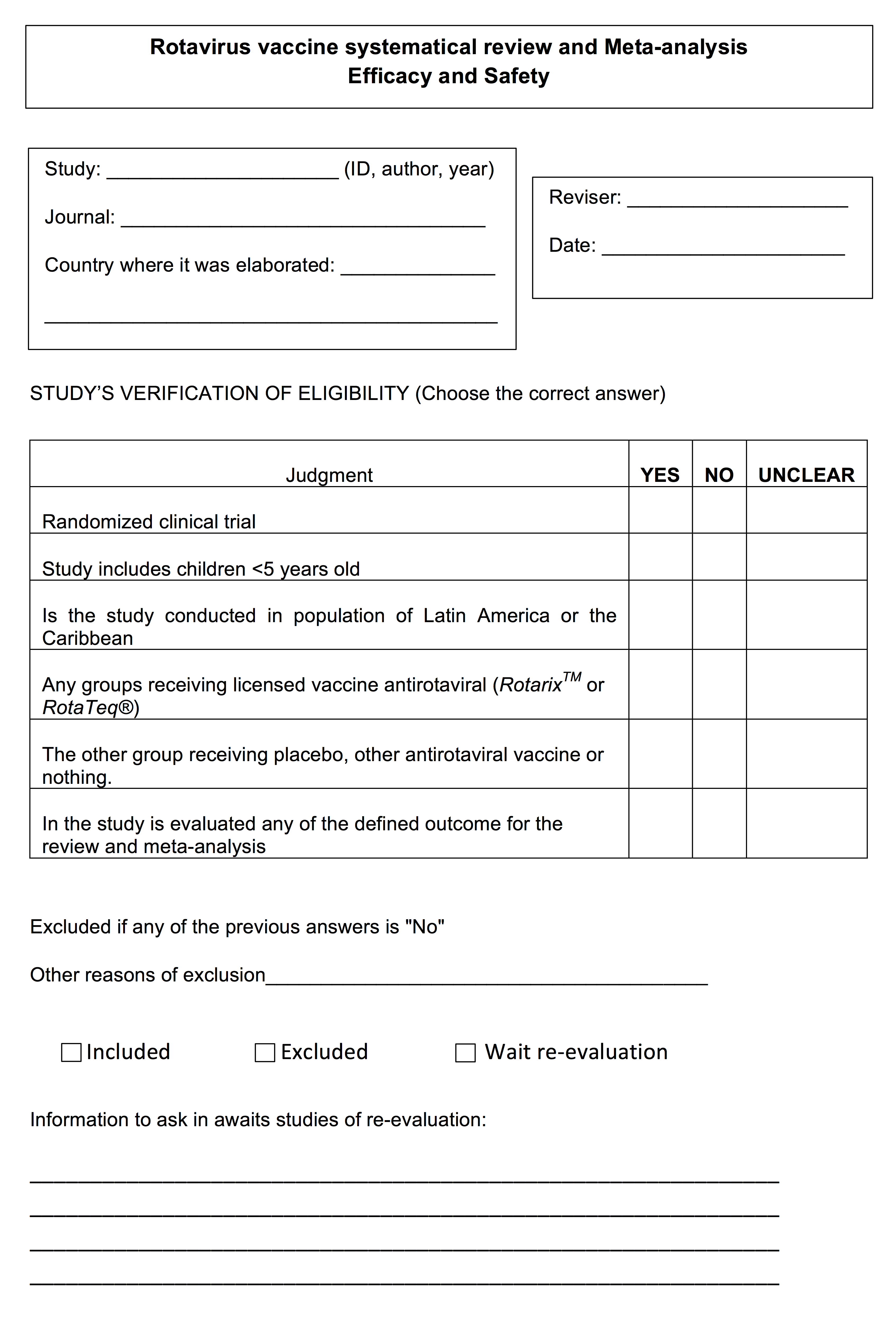


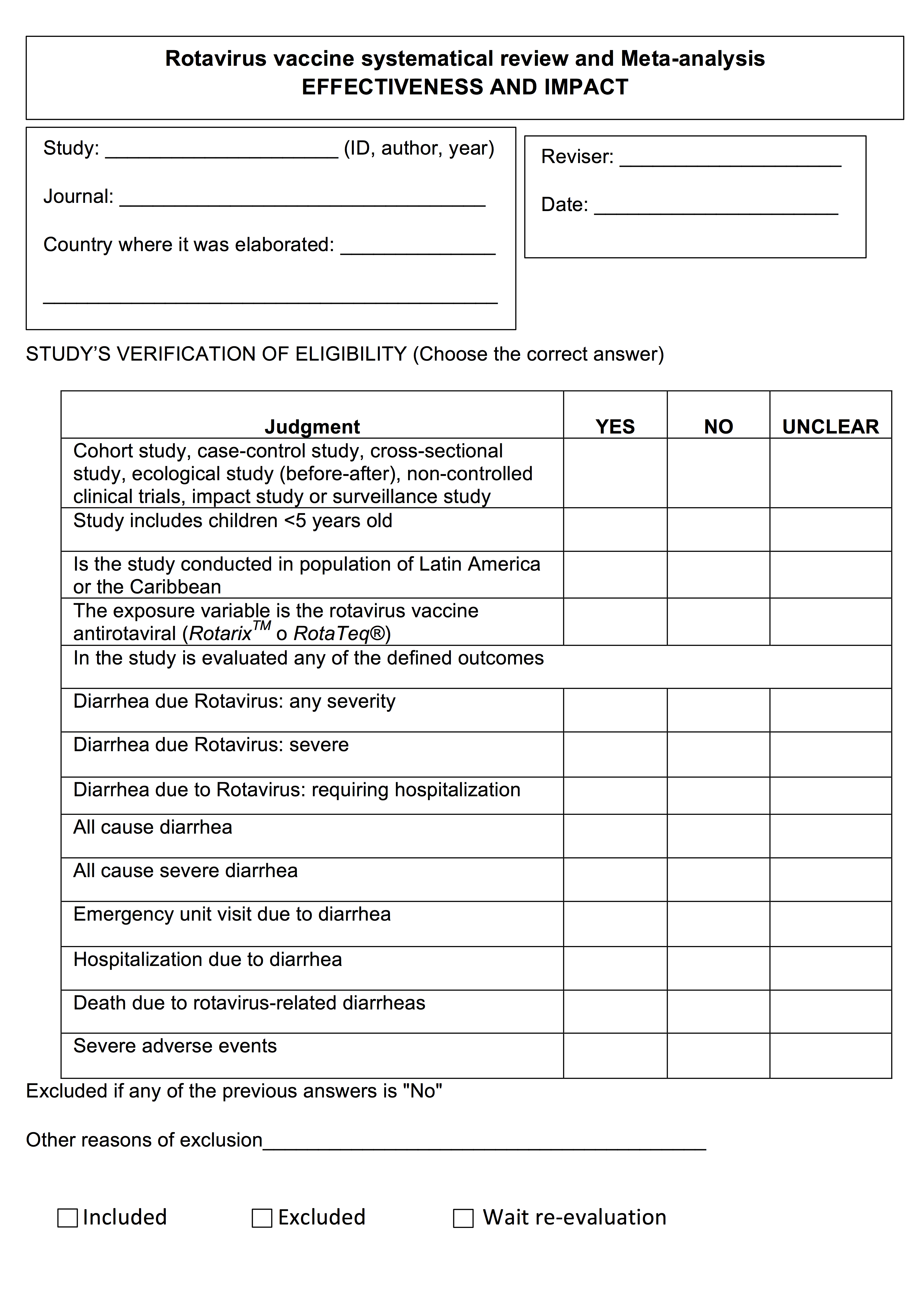


# Web-appendix 4. Extraction sheet for risk of bias assessment in trials evaluating efficacy/safety of rotavirus vaccine


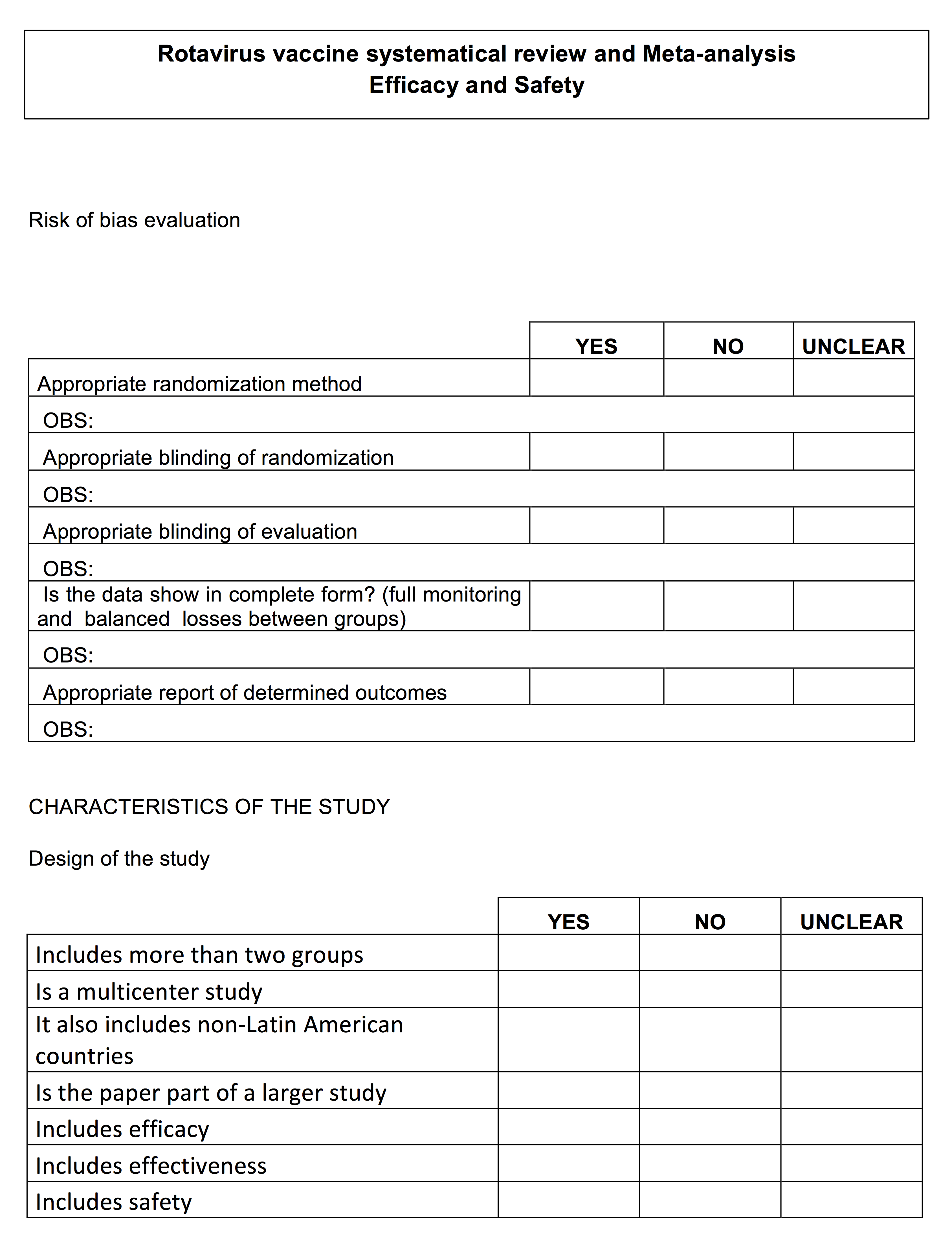


# Web-appendix 5. Selected studies for efficacy/safety evaluation of rotavirus vaccine

Selected studies after applied PRISMA* flow chart for the systematic review to evaluate rotavirus vaccine efficacy and safety in countries from Latin America and the Caribbean.

References of the included trials

Araujo EC, Clemens SAC, Oliveira CS, Justino MCA, Rubio P, Gabbay YB, et al. Safety, immunogenicity, and protective efficacy of two doses of RIX4414 live attenuated human rotavirus vaccine in healthy Brazilian infants. J. Pediatr. (Rio. J). 2007;83:217–224.

Christie CDC, Duncan ND, Thame KA, Onorato MT, Smith HD, Malcolm LG, et al. Pentavalent rotavirus vaccine in developing countries: safety and health care resource utilization. Pediatrics. 2010;126:e1499–506

Linhares AC, Velázquez FR, Pérez-Schael I, Sáez-Llorens X, Abate H, Espinoza F, et al. Efficacy and safety of an oral live attenuated human rotavirus vaccine against rotavirus gastroenteritis during the first 2 years of life in Latin American infants: a randomised, double-blind, placebo-controlled phase III study. Lancet. 2008;371:1181–9.

Rojas OL, Caicedo L, Guzmán C, Rodríguez L-S, Castañeda J, Uribe L, et al. Evaluation of circulating intestinally committed memory B cells in children vaccinated with attenuated human rotavirus vaccine. Viral Immunol. 2007;20:300–11.

Ruiz-Palacios GM, Pérez-Schael I, Velázquez FR, Abate H, Breuer T, Clemens SC, et al. Safety and efficacy of an attenuated vaccine against severe rotavirus gastroenteritis. N. Engl. J. Med. 2006;354:11–22.

Ruiz-Palacios GM, Guerrero ML, Bautista-Márquez A, Ortega-Gallegos H, Tuz-Dzib F, Reyes-González L, et al. Dose response and efficacy of a live, attenuated human rotavirus vaccine in Mexican infants. Pediatrics. 2007;120:e253–61

Salinas B, Pérez Schael I, Linhares AC, Ruiz Palacios GM, Guerrero ML, Yarzábal JP, et al. Evaluation of safety, immunogenicity and efficacy of an attenuated rotavirus vaccine, RIX4414: A randomized, placebo-controlled trial in Latin American infants. Pediatr. Infect. Dis. J. 2005;24:807–16

Tregnaghi MW, Abate HJ, Valencia A, Lopez P, Da Silveira TR, Rivera L, et al. Human rotavirus vaccine is highly efficacious when coadministered with routine expanded program of immunization vaccines including oral poliovirus vaccine in Latin America. Pediatr. Infect. Dis. J. 2011;30:e103–8

Vesikari T, Itzler R, Matson DO, Santosham M, Christie CDC, Coia M, et al. Efficacy of a pentavalent rotavirus vaccine in reducing rotavirus-associated health care utilization across three regions (11 countries). Int. J. Infect. Dis. 2007;11 Suppl 2:S29–35

# Web-appendix 6. Risk of bias graph

A) Graph illustrating the proportion of studies with judgments of risk of bias for each entry, and B) and risk of bias summary figure presenting study-specific judgment for each entry of included trials for efficacy/safety assessment of rotavirus vaccines in Latin America and the Caribbean. * **Risk of Bias:** High, clearly indicates bias in each domain; Low, clearly excludes bias in each domain**;** Unclear, insufficient information to permit judgment of risk of bias.

# Web-appendix 7. Summary of the characteristics of studies included for assessing efficacy/safety of rotavirus vaccines

# Web-appendix 8. Forest plot of meta-analysis for severe diarrhea of any cause

Rotavirus immunization *vs.* placebo: relative risk for preventing severe diarrhea of any cause.

# Web-appendix 9. Forest plot of meta-analysis for treatment-related mortality

Rotavirus immunization *vs.* placebo: association between death and treatment

# Web-appendix 10. Forest plot of meta-analysis for treatment-related intussusception

Rotavirus immunization *vs.* placebo: association between intussusception and treatment.

**Web-appendix 11**. Common severe adverse events occurring in rotavirus immunized and placebo groups.

# Web-appendix 12. Forest plot of meta-analysis for treatment-related severe adverse events

Rotavirus immunization *vs.* placebo: association between severe adverse events and treatment.

# Web-appendix 13. Identified studies for effectiveness evaluation of rotavirus vaccine

Case-control studies selected after applied PRISMA* flow chart for the systematic review to evaluate rotavirus vaccine effectiveness in countries from Latin America and the Caribbean.

| RotaTeq^®^ |  |  |
| --- | --- | --- |
| Rotarix^TM^ |  |  |
|  | Analytical study | |
| *PRISMA = Preferred Reported Items for Systematic Reviews and Meta-Analysis | | |

References of the included trials

Correia JB, Patel MM, Nakagomi O, Montenegro FMU, Germano EM, Correia NB, et al. Effectiveness of monovalent rotavirus vaccine (RotarixTM) against severe diarrhea caused by serotypically unrelated G2P[4] strains in Brazil. J. Infect. Dis. 2010;201(3):363–9

Justino MCA, Linhares AC, Lanzieri TM, Miranda Y, Mascarenhas JDP, Abreu E, et al. Effectiveness of the monovalent G1P[8] human rotavirus vaccine against hospitalization for severe G2P[4] rotavirus gastroenteritis in Belém, Brazil. Pediatr. Infect. Dis. J. 2011;30:396–401.

De Palma O, Cruz L, Ramos H, de Baires A, Villatoro N, Pastor D, et al. Effectiveness of rotavirus vaccination against childhood diarrhoea in El Salvador: case-control study. BMJ. 2010;340:c2825

Patel M, Pedreira C, De Oliveira LH, Tate J, Orozco M, Mercado J, et al. Association between pentavalent rotavirus vaccine and severe rotavirus diarrhea among children in Nicaragua. JAMA. 2009;301:2243–51.

# Web-appendix 14. Summary of the characteristics of studies included for evaluate effectivenes of rotavirus vaccine

# Web-appendix 15. Summary of results of studies assessing effectiveness of rotavirus vaccine
